# A genome-wide signature of glucocorticoid receptor binding in neuronal PC12 cells

**DOI:** 10.1186/1471-2202-13-118

**Published:** 2012-10-03

**Authors:** J Annelies E Polman, Jennifer E Welten, Danny S Bosch, Robert T de Jonge, Judit Balog, Silvère M van der Maarel, E Ronald de Kloet, Nicole A Datson

**Affiliations:** 1 Division of Medical Pharmacology, Leiden/Amsterdam Center for Drug Research, Leiden University Medical Center, Leiden, 2333 CC, the Netherlands; 2 Department of Human Genetics, Leiden University Medical Center, Leiden, 2333 ZC, the Netherlands

**Keywords:** Glucocorticoid receptor, Neuronal context, Glucocorticoid response element, PC12 cells, ChIP-Seq

## Abstract

**Background:**

Glucocorticoids, secreted by the adrenals in response to stress, profoundly affect structure and plasticity of neurons. Glucocorticoid action in neurons is mediated by glucocorticoid receptors (GR) that operate as transcription factors in the regulation of gene expression and either bind directly to genomic glucocorticoid response elements (GREs) or indirectly to the genome via interactions with bound transcription factors. These two modes of action, respectively called transactivation and transrepression, result in the regulation of a wide variety of genes important for neuronal function. The objective of the present study was to identify genome-wide glucocorticoid receptor binding sites in neuronal PC12 cells using Chromatin ImmunoPrecipitation combined with next generation sequencing (ChIP-Seq).

**Results:**

In total we identified 1183 genomic binding sites of GR, the majority of which were novel and not identified in other ChIP-Seq studies on GR binding. More than half (58%) of the binding sites contained a GRE. The remaining 42% of the GBS did not harbour a GRE and therefore likely bind GR via an intermediate transcription factor tethering GR to the DNA. While the GRE-containing binding sites were more often located nearby genes involved in general cell functions and processes such as apoptosis, cell motion, protein dimerization activity and vasculature development, the binding sites without a GRE were located nearby genes with a clear role in neuronal processes such as neuron projection morphogenesis, neuron projection regeneration, synaptic transmission and catecholamine biosynthetic process. A closer look at the sequence of the GR binding sites revealed the presence of several motifs for transcription factors that are highly divergent from those previously linked to GR-signaling, including Gabpa, Prrx2, Zfp281, Gata1 and Zbtb3. These transcription factors may represent novel crosstalk partners of GR in a neuronal context.

**Conclusions:**

Here we present the first genome-wide inventory of GR-binding sites in a neuronal context. These results provide an exciting first global view into neuronal GR targets and the neuron-specific modes of GR action and potentially contributes to our understanding of glucocorticoid action in the brain.

## Background

The brain is a major target of glucocorticoids (GCs) that are secreted by the hypothalamus-pituitary-adrenal axis in response to stress. In the brain there are two receptors for glucocorticoids, the mineralocorticoid receptor (MR) and the glucocorticoid receptor (GR), that differ in their expression pattern and affinity for GCs. GR is abundantly expressed throughout the brain both in neurons and non-neuronal cells such as microglia and astrocytes [1-4]. GR has a relatively low affinity for its ligand, cortisol in humans and corticosterone in rodents (both abbreviated as CORT), and is activated when CORT levels rise, for example during stress. Upon CORT binding, GR migrates from the cytoplasm to the nucleus where it is involved in the regulation of gene transcription.

Transcriptional regulation by GR is complex and several molecular mechanisms have been described involving both homodimers and monomers of GR. Direct binding of GR dimers to Glucocorticoid Response Elements (GREs) in the vicinity of target genes, a process known as transactivation, is the classical mode of action which generally results in a potentiation of transcription [5]. However, GR also exhibits extensive crosstalk with other transcription factors (TFs), and besides simple GREs composite sites exist that contain a binding site for another TF in close proximity to the GRE, resulting in either a synergistic activation or a repression of transcription [6,7]. Furthermore, GR monomers can also exert effects on gene transcription by indirectly binding to the DNA via an intermediate DNA-bound TF in so called tethering response elements [8], mostly resulting in a repression of transcription of the associated gene, a process referred to as transrepression. This extensive crosstalk of GR with other TFs not only vastly expands the range of GR-control on physiological processes compared to the classical GRE-driven transcriptional control in simple GREs, but it also underlies the highly context-dependent action of GCs.

Several TFs have been described that participate in this crosstalk with GR, including Oct1, Ets1, AP-1 and CREB at composite GREs and NF-κB, AP-1, CREB, Oct-1/2, STAT6, SMAD3,4 and PU.1/Spi-1 at tethering sites [6,7,9-16]. However, most of these crosstalk partners of GR have been identified in studies on the immunosuppressive and the tumor suppressor properties of GR [17-19], while very little is known about crosstalk partners in a neuronal context.

In neuronal cells GR regulates the expression of a wide diversity of genes involved in general cellular processes such as energy metabolism, cell cycle and response to oxidative stress, but also clearly is involved in regulating a wide variety of genes important for neuronal structure and plasticity [20]. Despite the fact that many neuronal GC-responsive genes have been identified [21-23], it remains unclear whether these genes are primary or downstream targets of GR. The onset of high-throughput sequencing combined with chromatin immunoprecipitation (ChIP-Seq) has made it possible to characterize genome-wide binding sites of TFs and today several studies have used this approach to identify global primary GR-targets in a variety of cell types, including human lung carcinoma cells (A549), mouse adipocytes (3T3-L1), premalignant breast epithelial cells (MCF10A-Myc), murine mammary epithelial cells (3134) and pituitary (AtT-20) cells [24-27]. These studies have yielded an unprecedented insight into genome wide GR targets as well as molecular mechanisms of GR-signaling, but perhaps one of the most striking findings is the low degree of overlap in GR binding sites when comparing different cell types, indicating that GR occupancy is highly cell type specific [25]. Therefore, in order to gain insight into global GR primary target in neurons, it is essential to characterize GR binding in a neuronal context. So far no studies have taken a ChiP-Seq approach to characterize GR-binding in a neuronal context.

The aim of the current study was to analyze genome-wide GR-binding sites (GBS) in rat neuronal PC12 cells using ChIP-Seq. The PC12 cell line is derived from a pheochromocytoma of the rat adrenal medulla and can be differentiated into a neuronal phenotype by stimulation with nerve growth factor [28]. NGF- treated PC12 cells stop dividing, develop neurites, display electrical activity and develop many other properties similar to those of sympathetic neurons [28,29]. They are considered a useful model system for neurosecretion and neuronal differentiation [30] and have been extensively used to study neuronal function in relation to GCs [31-33]. In this study, besides identifying the binding sites of GR in neuronal PC12 cells, we analysed which genes were located in the vicinity of the binding sites, which gene ontology classes were overrepresented, whether GR-binding resulted in regulation of gene expression of nearby genes and the motif composition of the binding sites.

## Results

### Identification and genomic distribution of GR binding sites in PC12 cells

**Figure 1:**
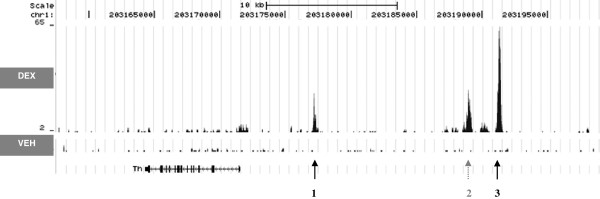
**Genomic distribution of Glucocorticoid Receptor binding sites (GBS) upstream of the Tyrosine Hydroxylase gene (TH).** Two significant peaks representing GR-binding are observed at approximately 5.7 kb (peak 1) and 19.7 kb (peak 3) upstream of the transcription start site (TSS) as indicated by arrows. The 5.7 kb GBS was previously described in PC12 cells transfected with the TH promoter [34]. A third peak (peak 2) upstream of the TH gene was apparent, but was not significantly above background (IgG signal) at this position, so was not further analysed. Data was visualized with the UCSC genome browser [35].

**Figure 2:**
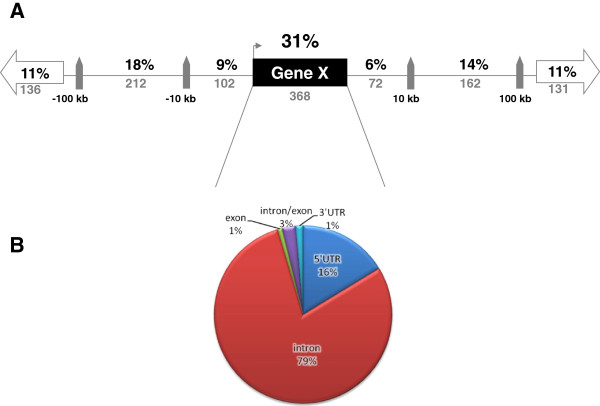
**Genomic distribution of GBS relative to nearby genes.A** The percentage of GBS that are present intragenically or within a certain range from the nearest gene are indicated, showing that the number of GBS located within a gene is highest. **B** Intragenic GBS can be further subdivided into subregions: 5’UTR (exon or intron), intron, exon, intron/exon overlap and 3’UTR regions.

ChIP-Seq resulted in the identification of 2,252 genomic regions that were bound by GR after 90 minutes of continuous DEX-stimulation of neuronal PC12 cells. Of this list, 1,183 regions had a p-value <0.05 and were considered to be significant and were used for further analysis. An example of the ChIP-Seq data showing GR-binding upstream of the tyrosine hydroxylase gene (Th) is shown in Figure 1. To get insight into the genomic distribution of GR binding, the shortest distance of the center of each significant GBS to the nearest gene was determined within a 100 kb region. Approximately one third (31%) of all significant GBS was located within a gene, while 47% did not overlap with a gene but were located within a 100 kb distance upstream or downstream of a gene (Figure 2A). The remaining 22% of GBS were located further than 100 kb upstream or downstream from the closest gene. In total there were more GBS located upstream to genes than downstream: 38% vs 31% respectively.

Based on their genetic location, the intragenic GBS were subdivided into the following groups: 5’UTR and 3’UTR (including introns and exons that are located there), introns, exons and GBS overlapping an exon/intron junction (Figure 2B). The majority (79%) of intragenically located GBS were confined to intronic regions. Only 16% of the intragenic GBS were located within the 5’UTR, upstream of the coding sequence of the gene, a region classically considered to be involved in regulation of gene expression [36]. A list of the 50 most significant regions containing GBS and the most nearby gene is shown in Table 1. The full list of 1,183 GBS is available in the additional material (Additional file 1: Table S1).

**Table 1:** Top 50 of significance GR-binding sites

**Region**	**Chr**	**Start**	**End**	**p-value**	**Nearest TS (bp)**	**Gene symbol**	**Gene description**
1	chr14	92730207	92730300	0	58382	ddc	dopa decarboxylase
2	chr12	4225730	4225800	0	41349	N4BP2L2	NEDD4 binding protein 2-like 2
3	chr8	84942839	84942945	6.66E-16	−40027	FILIP1	filamin A interacting protein 1
4	chr3	159002303	159002377	3.44E-15	−68132	ptpn1	protein tyrosine phosphate, non-receptor type 1
**5**	**chr1**	**170271212**	**170271308**	**1.07E-14**	**38820**	**PARVA**	**parvin, alpha**
6	chr20	44499841	44499940	1.25E-14	−27572	CDC2L6	cell division cycle 2-like 6(CDK8-like)
**7**	chr1	208485510	208485587	2.67E-14	24399	FRMD8	FERM domain containing 8
8	chr10	55856373	55856471	1.24E-13	−3428	Per1	period homolog 1
9	chr10	95336367	95336493	1.82E-13	−1827	CYB561	cytochrome b-561
10	chr9	82139844	82139973	2.12E-13	−22571	AGFG1	ArfGAP with FG repeats 1
11	chr2	214126583	214126707	5.58E-13	119898	snx7	sorting nexin 7
**12**	**chr7**	**71292501**	**71292588**	**1.31E-12**	**452930**	**Cohh1**	**Cohen syndrome homolog 1**
**13**	**chr10**	**108442435**	**108442545**	**1.13E-11**	**10507**	**Clqtnf1**	**C10q and tumor necrosis factor related protein 1**
**14**	**chr1**	**14981944**	**14982032**	**2.76E-11**	**16334**	**il20ra**	**interlukin 20 receptor, alpha**
**15**	**chr10**	**69940043**	**69940119**	**1.777E-10**	**250224**	**ACCN1**	**amiloride-sensitive cation channel 1, neuronal**
16	chr4	26732111	26732175	1.87E-10	−20533	cyp51	cytochrome P450, subfamily 51
17	chr1	203191165	203191336	2.39E-10	−19745	TH	tyrosine hydroxylase
18	chr3	159218917	159219016	2.51E-10	−11706	Pard6b	pa-6 (partitioning defective 6) homolog beta
**19**	**chr3**	**11220641**	**11220714**	**2.76E-10**	**11081**	**PPAPDC3**	**phosphatidic acid phosphatase type 2 domain containing 3**
20	chr13	98085339	98085424	8.73E-10	−40128	srp9	signal recognition particle 9
21	chr10	16132564	16132677	1.10E-09	−105921	LOC685957	cytoplasmic polyadenylation element binding protein 4
22	chr1	128794365	128794456	2.01E-09	−74086	CHD2	chromo domain helicase DNA binding protein 2
**23**	**chr1**	**148370534**	**148370610**	**2.72E-09**	**483095**	**Dlg2**	**discs, large homolog 2**
24	chr14	99179170	99179253	3.03E-09	−356843	etaa1	Ewing tumor-associated antigen 1; similar to ETAA16 protein
25	chr3	120538726	120538946	4.16E-09	-32899	CHGB	chromogranin B
26	chr16	36611065	36611165	5.78E-09	−285078	HAND2	heart and neural crest derivatives expressed 2
27	chr13	100632670	100632774	6.77E-09	58963	hlx	H2.0-like homeobox
28	chr1	83486283	83486376	8.07E-09	2797	ZFP36	zinc finger protein 36
29	chr2	242852558	242852668	1.23E-08	50854	sh3glb1	SH3-domain GRB2-like endophilin B1
30	chr19	36308488	36308565	1.45E-08	36674	Zfp90	zinc finger protein 90
31	chr2	218383549	218383633	1.75E-08	12542	F3	coagulation factor III (thromboplastin, tissue factor)
32	chr	72391772	72391896	2.24E-08	−85708	YWHAZ	tyrosine 3-monooxygenase/tryptophan 5-monooxygenase activation protein, zeta polypeptide
33	chr10	63291887	6321961	2.45E-08	−26356	rph3al	rabphilin 3A-like (without C2 domains)
34	CHR1	203177190	203177316	3.19E-08	−5747	TH	tyrosine hydroxylase
35	chr8	121454771	121454890	4.05E-08	−35296	Snrk	SNF related kinase
**36**	**chr17**	**7534539**	**7534637**	**4.25E-08**	**90348**	**Npepo**	**aminopeptidase O**
**37**	**chr7**	**45295426**	**45295501**	**4.78E-08**	**155311**	**PPFIA2**	**protein tyrosine phosphatase, receptor type, f polypeptide (PTPRF), interacting protein (liprin), alpha**
38	chr10	19716893	19717068	6.83E-08	−7100	ccdc99	coiled-coil domain containing 99
**39**	**chr1**	**179785291**	**179785400**	**7.20E-08**	**17710**	**Polr3e**	**polymerase (RNA) III (DNA directed) polypeptide E (80kd)**
**40**	**chr13**	**85831724**	**85831796**	**7.86E-08**	**14876**	**DDR2**	**discoid in domain receptor tyrosine kinase 2**
41	chr2	240315238	240315326	8.89E-08	−18368	PDLIM5	PDZ and LIM domain 5
42	chr3	155445628	155445734	1.01E-07	−503522	Sdc4	syndecan 4
43	chr16	22356139	22356299	1.05E-07	19144	SLC18A1	solute carrier family 18 (vesicular monoamine), member 1
44	chr10	75271499	75271582	1.65E-07	−112174	LOC688105	hypothetical protein LOC688105; LOC360590
**45**	**chr16**	**6562495**	**6562603**	**1.71E-07**	**2931**	**NT5DC2**	**5’-nucleotidase domain containing 2**
**46**	**chr16**	**22377983**	**22378135**	**1.74E-07**	**21756**	**SLC18A1**	**solute carrier family 18 (vesicular monoamine), member 1**
**47**	**chr6**	**75731811**	**75731965**	**1.97E-07**	**587**	**NFKBIA**	**nuclear factor of kappa light polypeptide gene enhancer in B-cells inhibitor, alpha**
48	chr8	68790635	68790830	2.10E-07	−84	Rab11a	RAB11a, member RAS oncogene family
49	chr8	44864109	44864180	3.27E-07	188782	SORL1	sortilin-related receptor, LDLR class A repeats-containing
50	chr8	124992930	124993041	4.02E-07	237699	Cx3cr1	chemokine (C-X3-C motif) receptor 1

The 50 most significant GR-binding sites (GBS) as determined by CLCbio workbench software. Per GBS, the p-value is indicated as well as the nearest gene and the distance relative to the Transcription Start Site (TSS) of this gene. Negative numbers indicate a location upstream of the TSS, positve numbers downsteam of the TSS. GBS that are located intragenically are indicated in bold print.

### Reliability of ChIP-Seq data

**Figure 3:**
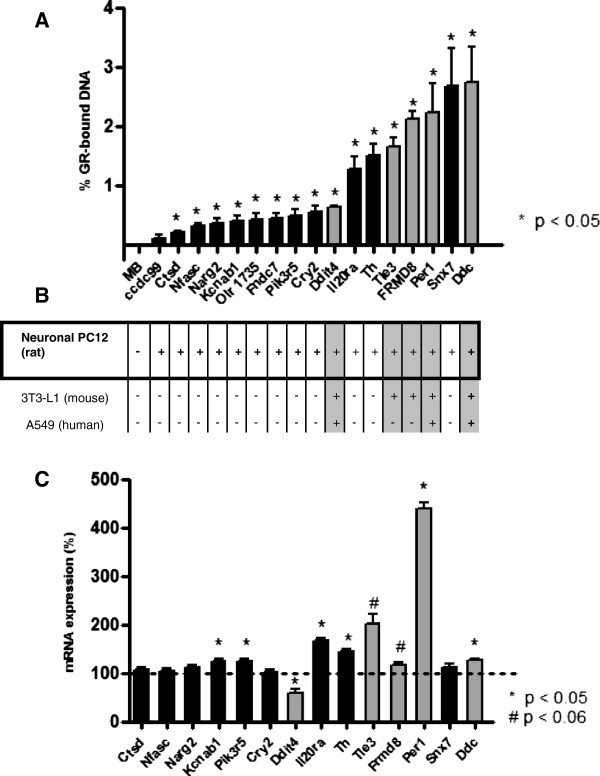
**Validation of GR binding sites and effects on mRNA expression. A** ChIP-PCR validation of identified GBS, previously shown to be GR-targets in literature (grey bars) or representing newly identified GBS (black bars). The genes that are associated with the GBS are listed on the x-axis. The y-axis represents the % of input DNA that was bound by the GR after subtracting the aspecific IgG-bound fraction and the amount of GR bound after vehicle (VEH) treatment. The error bars represent the standard error of the mean (SEM) when comparing the DEX-induced GBS versus the VEH-induced GBS. An unpaired two-tailed *T*-test was used for statistics. **B** Diagram indicating whether the known GR-binding regions were previously detected in other published GR-ChIPseq studies based on BlastZ-based interspecies conservation (http://main.g2.bx.psu.edu/) [37]. The genomic locations corresponding to the GBS are listed in Additional file 3: Table S3 as region numbers 1 (Ddc), 7 (FRMD8), 8 (Per1), 11 (Snx7), 14 (Il20ra), 17 (Th), 75 (TLE3), 94 (Ddit4), 345 (Olr1735), 352 (Fndc7), 366 (Pik3r5), 526 (Cry2), 704 (Nfasc), 842 (Narg2), 976 (Kcnab1), 1020 (Ctsd). **C** mRNA expression of the genes associated with the validated GBS after DEX-treatment relative to VEH-treatment (100%). Expression was normalized against tubulin 2a mRNA expression. The non-parametric Wilcoxon Signed Ranks Test was used for statistics.

To assess the reliability of the ChIP-seq data and the stringency of the applied statistical threshold, ChIP-qPCR experiments were performed in a new isolate of GR-bound DNA on a total of 17 GBS which covered a wide range of p-values (from 1E-6 to 0.03). The selection included five significant regions previously identified in other studies, in the vicinity of Ddit4, Per1, Tle3, FRMD8 and Ddc, which were also identified in the current study and served as positive controls [26,27] Figure 3A, B: grey bars). In addition, 12 novel GBS identified in this study in neuronal PC12 cells were selected for validation (Figure 3A: black bars). All but one GBS (Ccdc99) were successfully validated, showing that the selected cut-off of significant GBS (p-value < 0.05) was appropriate. Several of the novel GBS identified and validated in neuronal PC12 cells were associated with genes that have a known neuronal function, such as dopamine decarboxylase (Ddc) and tyrosine hydroxylase (TH), both important enzymes in the biosynthesis of catecholamines. Other examples are voltage-gated potassium channel subunit beta-1 (Kcnab1), NMDA receptor-regulated gene 2 (Narg2), Period circadian protein homolog 1 (Per1) and neurofascin (Nfasc).

### GR-binding sites and regulation of nearby genes

RNA was isolated from neuronal PC12-cells to establish whether GR activation by DEX-treatment induced expression of the genes closest to the validated GBS. Six out of 14 genes (Per1, Ddc, Kcnab1, Pik3r5, Il20ra, Th) showed a significant upregulation upon GR activation and another 2 genes (Frmd8 and Tle3) a clear trend towards significance with p-values of 0.055 and 0.051 respectively (Figure 3C). One gene, Ddit4, was downregulated by GR activation rather than upregulated. Five out of the 14 genes tested did not show a change in expression at the time point measured, i.e. 3 hours after GR activation. Eight out of 14 tested genes contained a GRE, including the GBS near Ddit4.

### Overlap with GR-binding sites in other tissues is limited

**Figure 4:**
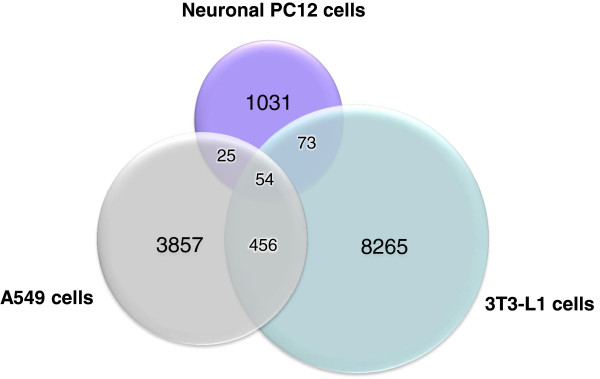
**Venn diagram representing overlap between GR-targets in different ChIP-Seq studies.** The overlap of GBS identified in PC12 cells is compared to those genomic regions bound by GR in two other ChIP-Seq studies in human lung carcinoma cells (A549) [26] and mouse adipocytes (3T3-L1) [27].

We next compared the GR binding regions in rat neuronal PC12 cells to two previously published GR ChIP-Seq studies performed in human lung carcinoma (A549) [26] and mouse adipocytes (3T3-L1) [27]. This resulted in a list of GBS unique to neuronal PC12 cells and lists of GBS shared with either or both of the other cell types. The majority of GBS identified in PC12, 1,031 in total, appeared unique to neuronal PC12 cells. Only 79 (7%) of the GBS identified in PC12 cells were shared with A549 cells and 127 (11%) with 3T3-L1 cells (Figure 4). A similar degree of overlap was observed comparing GBS of A549 and 3T3-L1 cells, that shared a total of 510 GBS being 12% and 6% respectively. Only 54 GBS (4%) of all PC12 GBS were common to all 3 cell types.

### PC12-specific GBS are located nearby genes with a neuronal function

To analyse which biological processes are likely to be affected by GR-binding in neuronal PC12 cells, the genes nearest to the GBS were analysed for overrepresentation of specific gene ontology classes using DAVID [38,39]. Genes closest to 1,031 sites uniquely identified in PC12 cells were used as input in the analysis. The genes near PC12-unique GBS had a high representation of GO-terms linked to neuronal function and clustering of all identified GO-terms revealed that the most enriched cluster in this group was “neuron development”, with other neuron-related clusters being “neuron projection”, “synapse” and “biogenic amine biosynthetic process” (Table 2).

**Table 2:** Top 10 enriched functional GO clusters in neuronal PC12-specific GR binding regions (GBS)

	**Neuronal PC212 unique GBS**		
	**GO Term**	**Category**	**Enrichment score**
1	neuron development	BP	4.4
2	cytoplasmic vesicle	CC	3.4
3	neuron projection	CC	3.1
4	metal ion binding	MF	3.0
5	blood vessel development	BP	3.0
6	cell motion	BP	2.8
7	identical protein binding	MF	2.6
8	biogenic amine biosynthetic process	BP	2.6
9	synapse	CC	2.2
10	protein tyrosine kinase activity	MF	2.0

Gene ontology analysis of genes associated with GBS identified in neuronal PC12 cells. The 10 most enriched functional GO clusters in GBS that are uniquely found in neuronal PC12 cells. Analysis was performed with the Database for Annotation, Visualization and Integrated Discovery (DAVID). Per cluster, the first GO-term is shown. In addition, the category to which the GO term belongs to is indicated, i.e. Biological Processes (BP), Molecular Function (MF) or Cellular Compartment (CC). The enrichment score indicates the geometric mean (in -log scale) of p-value of the GO cluster.

These results indicate that in neuronal PC12 cells the majority of GR binding is to genomic regions that are nearby or within genes with a known neuronal function. The full list of GO terms of the genes associated with the PC12-unique GBS are available in the additional material (Additional file 2: Table S2).

### GR binding sites represent both transactivation and transrepression modes of action

**Figure 5:**
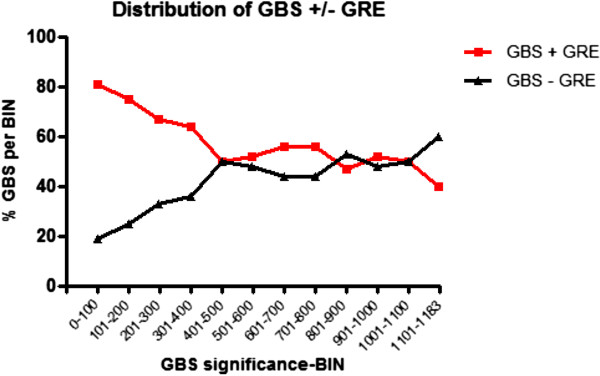
**Significance of GR-binding sites with and without a GRE.** GR binding sites containing a GRE-like sequence have smaller p-values in the ChIP-Seq data compared to those regions without a GRE. On the x-axis the 1183 GBS are ranked into BINs consisting of 100 binding regions ranked according to significance. For example, the 100 most significant GBS are represented in BIN 1 (1–100), while the 83 least significant GBS are represented in the last BIN (1101–1183). On the y-axis the percentage of GRE and non-GRE containing GBS per BIN is indicated.

Screening the significant GBS with MEME and TOMTOM for presence of known DNA-motifs revealed that 683 (58%) regions contained a Glucocorticoid Response Element (GRE). The identified GRE-motif was similar to the motif identified by others and also had a comparable prevalence [25,26]. This indicates that more than half of the GBS are most likely involved in transactivational effects of GR on gene transcription. We subsequently subdivided the list of GBS into a group of GBS with GREs, in which GR presumably exerts its actions via transactivation and the remainder without GREs, in which GR in all probability operates via transrepression of other transcription factors. Strikingly, the most significant GBS were enriched for GREs, while non-GRE containing GBS tended to have a lower p-value in the ChIP-Seq data (Figure 5). More than 80% of the top 100 most significant GBS contained a GRE, dropping to approximately 50% for GBS ranking lower in the list from position 400 downwards.

**Figure 6:**
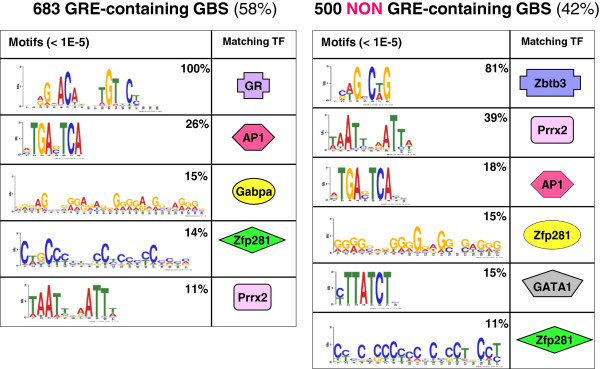
**MEME de novo motif discovery within GBS. A**. Motifs for transcription factor binding in the 683 GBS that contain a GRE-like sequence. **B**. Motifs for transcription factor binding in the 500 GBS without a GRE. Analysis was performed within a 200 bp-frame containing the GBS-centre in the middle. The frequency of identified motifs in the PC12-dataset is indicated as well as transcription factors of which the known binding motif most significantly matches the identified motif. Only motifs with an E-value < 1E-5 are depicted.

Not only the significance of the GBS differed between GRE and non-GRE containing binding sites, but also their composition in terms of motifs for transcription factor binding differed considerably. Both groups were subjected to de novo motif discovery to investigate the prevalence and identity of other motifs representing transcription factor binding sites within the binding regions. A total of 225 (33%) of the 683 GRE-containing GBS represented simple GREs, only harbouring a GRE-like sequence but no other motifs (Figure 6). However, the majority of the GRE-containing GBS represented so called composite sites and also contained one or more other motifs besides the GRE. In the group of GRE-containing genomic regions a motif for binding of Activator Protein-1 (AP-1) was most frequently observed, followed by motifs for binding of GA binding protein transcription factor, alpha subunit (Gabpa), Zinc Finger Protein 281 (Zfp281) and paired related homeobox 2 (Prrx2) (Figure 6). An entirely different distribution of motifs was observed in the genomic regions that did not contain a GRE. Interestingly, two motifs were identified that were unique for the regions without a GRE: a motif for binding of zinc finger and BTB (bric-a-bric, tramtrack, broad complex)-domain- containing 3 (ZBTB3) gene, present in over 80% of the regions, and a motif for binding of GATA binding protein 1 (GATA1), present in 15% of the genomic regions (Figure 6). Besides differences there were also some motifs found in both groups, regardless of whether the regions contained a GRE or not. For example, in both groups motifs corresponding to AP-1, Prrx2 and Zfp281 were identified, albeit at different frequencies.

**Figure 7:**
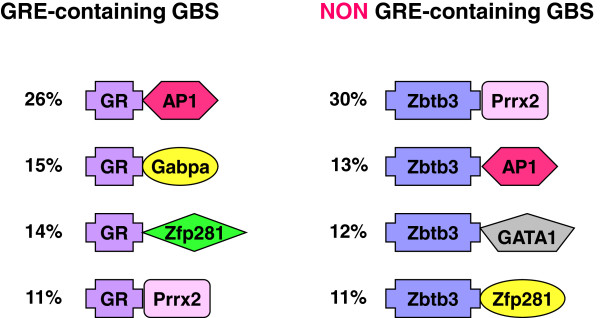
**The most frequent motif-combinations within the GBS.** Specific combinations of motifs for transcription factor binding were observed, with differences in occurrence and frequency between the GBS with and without a GRE. TF: transcription factor.

Next, the co-occurrence of the various motifs was investigated. In the GRE-containing group, 26% of the GBS contained an AP-1 site besides a GRE, making it the most prevalent combination of transcription factor binding sites. Other frequently observed combinations of motifs were a GRE in conjunction with motifs for binding of Gabpa, Zfp281 and Prrx2 (Figure 7). In the group without a GRE, all frequently observed combinations of motifs included Zbtb3. The most frequently observed combination was Zbtb3 in conjunction with Prrx2 (in 30% of the regions), followed by combinations of Zbtb3 with AP-1, GATA1 and Zfp281.

### Different biological processes are regulated via transactivtion and transrepressive modes of action

We subsequently investigated whether GBS that contain a GRE regulated different biological processes than those without a GRE, representing transactivation or transrepression modes of action respectively. Genes near GRE-containing GBS showed an involvement in general cell functions and processes such as apoptosis, cell motion, protein dimerization activity and vasculature development (Table 3). In contrast, genes near regions without a GRE had a clear role in neuronal processes such as neuron projection morphogenesis, neuron projection regeneration, synaptic transmission and catecholamine biosynthetic process. The full list of GO terms of the genes associated with GBS with and without GREs are available in the additional material (Additional file 3: Table S3).

**Table 3:** Top 10 enriched functional GO clusters in GR binding regions(GBS) with and without a GRE

	**GBS with GRE**			**GBS without GRE**		
	**GO Term**	**Category**	**Enrichment score**	**GO Term**	**Category**	**Enrichment score**
1	cell motion	BP	4.2	neuron projection morphogenesis.	BP	3.8
2	protein kinase binding	MF	3.5	cytoplasmic vesicle	CC	2.5
3	vasculature development	BP	3.3	metal ion binding	MF	2.4
4	protein dimerization activity	MF	2.9	phospholipid binding	MF	2.3
5	metal ion binding	MF	2.8	catecholamine biosynthetic process	BP	2.2
6	regulation of apoptosis	BP	2.3	protein complex assembly	BP	2.0
7	apoptosis	BP	2.2	muscle cell development	BP	1.9
8	regulation of myeloid cell differentiation	BP	2.1	neuron projection regeneration	BP	1.6
9	cell adhesion	BP	1.9	actin filament binding	MF	1.6
10	cytoplasmic vesicle	CC	1.8	synaptic transmission	BP	1.6

The 10 most enriched functional GO clusters in GBS that do or do not contain a GRE according to the Database for Annotation, Visualization and Integrated Discovery (DAVID). In both cases the GO-term that best represents the annotation cluster is shown. In addition, the category to which the GO term belongs to is indicated, i.e. Biological Processes (BP), Molecular Function (MF) or Cellular Compartment (CC). The enrichment score indicates the geometric mean (in -log scale) of p-value of the GO cluster.

## Discussion

GR is widely expressed throughout body and brain and is an important transcriptional regulator of a diversity of biological processes, ranging from glucose and lipid homeostasis to immune suppression and cell proliferation and differentiation. Today several ChIP-Seq studies have been published focusing on genome-wide discovery of GR binding in different cell types [24-27], and these studies have contributed immensely to our understanding of GR-signalling. What has become apparent, is that GR-binding is highly cell type-specific with minimal overlap in GBS between different cell types. Therefore, in order to gain insight into cell type-specific GR targets or mechanisms it is essential to investigate GR-signalling in a specific cell system or tissue of interest.

Here we present the first genome-wide discovery of GR-binding sites in a neuronal context. GR is an important transcription factor in neurons and is known to exert effects on neuronal structure and plasticity. So far the focus on GR-mediated action of glucocorticoids in a neuronal context has remained largely in the dark and most of the knowledge on GR modes of action, GR responsive genes and pathways and crosstalk partners of GR has come from studies on peripheral tissues including the immune system, the respiratory tract, skeletal muscle and adipose tissue as well as various types of cancer cells [40-42]. Approximately 1,100 genomic binding sites of GR were identified in neuronal PC12 cells, the majority of which are novel and display only very limited overlap with GR binding sites in other non-neuronal cell types. Moreover, most of the identified GR-binding sites were located in the vicinity of genes with a neuronal function. Finally, we identified several motifs for transcription factor binding that may represent novel crosstalk partners of GR in neurons.

### Reliability of ChIP-Seq data

We assessed whether our ChIP-Seq data met different reliability criteria. First, a very high proportion (16 out of 17 = 94%) of ChIP-Seq peaks covering a wide range of p-values could be validated using ChIP RT-qPCR in chromatin derived from an independent experiment. Second, several GBS were located in the vicinity of known GR-target genes. Third, 13% of the identified GBS overlapped with previously identified GBS in other tissues (mouse adipocytes and human lung carcinoma cells). Finally, highly significant motifs resembling GREs were detected in almost 60% of the peaks. Together these criteria underscore the high quality of our ChIP-Seq dataset of 1183 GBS.

### Genomic binding sites of GR by far exceed GR-responsive genes

The number of GBS identified in PC12 cells (1,183) was relatively low compared to other studies, i.e. 4,392 GBS in human lung carcinoma (A549) and 8,848 GBS in mouse adipocytes (3T3-L1) [25-27]. However, this could be the consequence of the high stringency we applied, supported by the high validation rate of GR-binding to 16 out of 17 selected GBS. We cannot exclude that the actual number of genomic regions exhibiting GR-binding in PC12 cells may be considerably higher. Comparison of GBS between different tissues is hampered by the different thresholds used in different studies without a standard accepted cut-off for reliability of ChIP-Seq data. Nonetheless, the identified GBS still considerably outnumbered by more than 10-fold the differentially expressed genes observed after a single 100 nM corticosterone pulse in neuronally differentiated PC12 cells [31]. In fact, this is a more general observation that applies to several of the ChIP-Seq studies on GR so far [25-27]. In A549 cells, for example, 1 hour of DEX-stimulation resulted in the identification of 4,392 GBS, whereas only 234 genes were differentially expressed at this time-point. Similarly in 3T3-L1 cells, 8,848 GBS were identified and 620 genes were found to be DEX-responsive after 6 hours. It therefore seems likely that GR-binding to genomic sites is a measure of the potential of GR to mediate effects on gene expression of nearby genes rather than a direct predictor of whether a gene is differentially expressed. Whether this potential is converted to an actual effect on transcription most likely depends to a large extent on the availability and binding of other TFs.

To further examine the relationship between GR-binding and regulation of gene expression of nearby genes, we tested whether GR activation by DEX regulated expression levels of the genes closest to the validated GBS. In approximately half of the cases we could validate differential mRNA expression of the associated genes, illustrating the functionality of GR-binding. This percentage is quite high, considering that for the tested genes the GBS were often located at large distances from the genes we tested and not necessarily in classical promoter regions. In a recent ChIP-Seq study on PAX8 binding sites the overlap with responsive genes as identified by DNA microarray was only 6.5%, despite the fact that only binding sites for PAX8 located within 1 kb of a TSS were taken into account [43].

However, this also means that in the other half of the cases we were not able to confirm an effect of GR-binding on expression of the closest gene. There are several possible explanations for this. First, maybe the nearest gene is not necessarily the most relevant gene for studying functional effects of GR-binding. Another explanation is that we measured gene expression at the wrong moment. Since GR binding precedes effects on gene expression, we chose to measure mRNA expression after 3 hours of DEX-exposure. We therefore cannot exclude that the genes that were not GC-responsive at this moment might still be regulated by GR, albeit at different time-points or under different conditions. Temporal dynamics of individual genes are known to differ [31,44,45], which may explain why not all genes with a nearby GBS are responsive to DEX at one given time-point. Investigating gene expression at other time-points would be necessary to determine this. In addition, measuring mRNA may not be sensitive enough to pick up the effects of GR-binding on gene expression in all cases. Conway-Campbell et al. showed that administration of pulses of corticosterone to adrenalectomised rats resulted in pulsatile GR-binding to the Per1 promoter region followed by a burst of transcription, which was measurable by qPCR of nascent heterogeneous nuclear RNA but was not obvious from measuring mRNA levels [45], despite the fact the Per1 is a well-established GR target gene. This may therefore also be the case for the genes in this study that showed no or a small change in expression 3 hrs after DEX administration. Finally, we can not exclude that some of the GBS are derived from unspecific binding at spurious genomic locations, due to the applied continuous dosing regime with the synthetic GC DEX, rather than pulsatile exposure to the endogenous ligand, explaining why differential expression of the nearest gene was not observed.

### Genomic location of GBS

What is becoming increasingly clear is that the majority of GBS are not located in promoter regions upstream of the transcription start site of genes or in the 5’UTR. In fact, only 9% of the significant GBS identified in the current study were located within 10kb upstream of the TSS and an additional 5% were located within the 5’UTR. In contrast, a higher number of GBS (11%) were located at a distance >100kb upstream the TSS. These distant regions might be functional, since it is known that transcription factor binding sites are able to exert effects on gene expression through chromosome folding and therefore can be effective at large genomic distances [46].

A much higher percentage of GBS occurred in intragenic regions, almost exclusively in introns, representing 31% of the total list of significant GBS. A similar phenomenon was observed in 3T3-L1 adipocytes, where 48% of the GBS were found in intragenic regions, either in exons or introns [27]. Why intragenic regions show so much GR binding is at the least intriguing. Studies using artificial constructs in luciferase reporter assays have suggested that intronic GBS contain GREs with functional properties [27].

The 1,183 GBS identified in this study were associated with considerably fewer than 1,183 different genes, given that there were many examples of multiple GBS being located in each others vicinity nearby the same gene. An example is Disks large homolog 2 (Dlg2), that had 7 different GBS located nearby or Tolloid-like protein 1 (TLL1) with 5 GBS nearby. A question that still needs answering is whether the most nearby gene to a GBS is in fact the most likely candidate to be transcriptionally regulated by GR binding, or whether multiple genes could be affected. Several GBS had multiple genes in their vicinity. To solve this point linking ChIP-Seq studies on TF-binding with expression studies remains important, as well as performing studies in which GBS are mutated in their natural chromatin environment to investigate the effect on transcription of nearby genes.

### Tissue-specificity of GR-binding reveals a neural signature

The majority of the GBS identified in this study were novel and unique to neuronal PC12 cells and were located nearby genes with a high representation of GO-terms linked to neuronal function. For example, one of the enriched GO clusters among the genes near PC12-unique GBS was “biogenic amine biosynthetic process”, which refers to the biosynthesis of biologically active amines, such as norepinephrine, histamine, and serotonin, many of which act as neurotransmitters. Indeed, we identified GBS in the vicinity of a number of genes involved in the synthesis of catecholamines, such as dopamine decarboxylase (Ddc) (Additional file 1: Table S1: regions nr. 17 and 34) and tyrosine hydroxylase (TH) (Additional file 1: Table S1: region 1). “Neuron projection” was another of the enriched GO clusters and accordingly several GBS were located in the vicinity of genes that play a role in outgrowth of axons, such as the semaphorins SEMA3E and SEMA5A (Additional file 1: Table S1: regions 529, 663, 774 and 891 respectively), proteins that act as axonal growth cone guidance molecules [47]. Four other GBS were located nearby SLIT2 (Additional file 1: Table S1: region 455) and SLIT3 (Additional file 1: Table S1: regions 53, 750 and 1134), molecules that act as guidance cues in cellular migration [48]. In addition, several GBS near genes involved in neurotransmission were observed, such as the serotonin receptors HTR1A, HTR1D, HTR1F and HTR2A (Additional file 1: Table S1: regions nr. 660, 284, 1070 and 807 respectively) and 18 GBS located near a wide variety of voltage-gated potassium channel subunits, including KCNA3, KCNA4, KCNAB1, KCNC1, KCNH1, KCNH2, KCNH6, KCNK9 and KCNMA1, which play a role in neuronal excitability and neurotransmitter release [49]. Finally, several GBS were located nearby the synaptotagmins SYT1 (Additional file 1: Table S1: region 752, 1011, 1159), SYT13 (Additional file 1: Table S1: region 1032) and SYT17 (Additional file 1: Table S1: region 280, 879, 1019) which are integral membrane proteins of synaptic vesicles thought to participates in triggering neurotransmitter release at the synapse [50]. These are just a few of the many examples of GBS located in the vicinity of genes with neuronal function. Tissue-specific co-factors or transcription factors likely mediate binding of GR to the DNA or alter chromatin accessibility, resulting in these distinct tissue-specific patterns of GR-binding.

The overlap in GBS with other tissues was low, with only 7% and 11% of the GBS overlapping with A549 cells 3T3-L1 cells respectively and is very much in line with what has been observed in expression studies and other GR ChIP-Seq studies. For example, comparison of mouse mammary and mouse pituitary cells revealed an overlap of 4.5% and 11.4% respectively of the total number of GBS identified in either of the cell types [25]. A similar high degree of tissue-specificity has also been observed for other TFs, such as STAT3, where an overlap of only 34 of 1352 (2.5%) identified STAT3 binding sites was observed when comparing ChIP-Seq data derived from 3 different tissues (mouse peritoneal macrophages. mouse embryonic stem cells and CD4+ T cells [51].

### Potential crosstalk partners of GR of relevance for neuronal function

GR operates in conjunction with an extensive network of other TFs. Previous studies in a non-neuronal setting, e.g. involving the immune system, muscle and adipose tissue, have generated extensive knowledge on GR-binding to the genome, the motifs that are recognized by GR and the transrepression partners that it can inhibit by protein-protein interaction [19,40-42]. However, confirmation of this knowledge in a neuronal context is lacking.

The importance of other TFs for GR-function is evident from the high percentage of GBS consisting of composite GREs or binding sites for multiple TFs we observed in this study. Only twenty percent of the identified GBS consisted of simple GREs, harbouring a GRE-like sequence but no other motifs. The vast majority of the GBS were composite sites containing binding motifs for multiple TFs. This included composite GREs that contained a GRE in addition to one or more other motifs, as well as tethering GBS that did not contain a direct binding site for GR but most often a combination of motifs for TF-binding. Motifs for binding of AP-1, were frequently observed in the GBS in PC12 cells, both in combination with a GRE as well as in tethering sites. AP-1 is a well-known crosstalk partner of GR [52] and AP-1 binding sites overlap extensively with GR binding sites [53]. Interestingly, however, we also observed a number of motifs for TFs within the GBS that may represent novel crosstalk partners of GR that are relevant in a neuronal context.

In composite GREs, besides AP-1, three different motifs were abundantly observed, corresponding to binding sites for Gapba, Zfp281 and Prrx2. In tethering sites, the most frequently observed motif was a binding motif for Zbtb3, occurring in more than 80% of the GBS and by far outnumbering AP-1 motifs which had a frequency of only 18%. Other abundant motifs represented binding sites for GATA1, Zfp281 and Prrx2. For many of these TFs information in literature is sparse. Moreover, a link to neuronal function and/or GR has not been reported.

Zfp281 is a GC-box binding transcription factor and is involved in the regulation of genes implicated in pluripotency of murine embryonic stem cells [54,55]. Recently, we identified GC-box associated motifs in flanking regions surrounding GREs of hippocampal CORT-responsive genes. The presence of a GC-box motif in close proximity to the GRE correlated with GR-binding in the hippocampus, but not in other non-neuronal cell types [56]. We hypothesized that GC-boxes may play a role in determining tissue specificity of GR binding to a defined group of GREs. The GC-box motif we identified in hippocampus resembled the binding motif of the MAZ TF which is in fact very similar to the Zfp281 motif identified in neuronal PC12 cells (Figure 6). According to Allen Brain Atlas [57], both MAZ and Zfp281 are very highly expressed in the mouse brain, especially in the hippocampus. Either one might be a novel crosstalk partner of GR in a neuronal context.

Gabpa, also known as nuclear respiratory factor 2 alpha (NRF2a), is a DNA-binding unit of the GA binding protein transcription factor which is involved in the nuclear control of mitochondrial function in neurons [58,59]. Gabpa responds to an altered energy demand within primary neurons by altering the expression of mitochondrial genes [60] and has been implicated in neuronal viability after brain injury [61]. Prrx2 is a member of the paired family of homeobox proteins, and is mainly known for its essential role in orofacial development [62]. It was recently discovered to be a novel pituitary transcription factor [63]. Otherwise very little is known on this TF and it has not been linked to GR-signalling before.

The transcription factor GATA1 is known to play an essential role in hematopoiesis [64]. GR was reported to interfere with GATA-1 function and inhibits the expression of erythroid structural genes [65]. Zbtb3 was observed in over 80% of the GBS that did not contain a GRE and was encountered in all frequently observed combinations of TFs binding sites in tethering GBS. Zbtb3 belongs to a family of transcription factors, many of which are important for B and T cell differentiation. A recent modeling study indicated that Zbtb3 may be a remote homologue of the Drosophila GAGA factor which is involved in both gene activation and gene repression and plays a role in the modulation of chromatin structure [66]. Zbtb3 contains a BTB domain, which plays a role in protein dimerization and transcriptional repression and interacts with histone deacetylase corepressor complexes such as NCoR (nuclear receptor corepressor) and SMRT (silencing mediator of retinoic acid and thyroid hormone receptor) [67-69]. Relevance for the brain has not been indicated yet.

It must be noted that linking the de novo motifs to binding sites of known proteins is difficult and since in many cases more proteins can bind to a given motif, additional ChIP-experiments would need to be performed to address experimentally whether the TFs described above and predicted by TOMTOM actually bind to the DNA at the identified genomic regions.

### Non-GRE containing tethering GBS are associated with genes involved in aspects of neuronal function

More than half of the GBS (58%) contained a GRE. Interestingly, the GRE-containing GBS were located near other types of genes than those without a GRE, as revealed by GO-analysis of the most nearby genes. While the GRE-containing GBS associated with more general cell functions such as apoptosis, cell motion, protein dimerization activity and vasculature development, the GBS without a GRE were more often located near genes involved in neuronal function. Motif analysis of the 54 sites in common between PC12, A549 and 3T3-L1 cells revealed that 91% contained a GRE (data not shown). This suggests that there is a core set of ubiquitous GBS that regulate key cellular processes in multiple tissues by the transactivation mode of action. On the other hand, tissue-specific TFs appear to play a role in tethering GR to genomic regions in a cell type-specific manner, regulating particular biological processes relevant for the tissue of interest. Of course many of the GRE-containing GBS were also unique to neuronal PC12 cells. In these cases it seems likely that tissue-specific TFs facilitate binding of GR to the chromatin, guiding it to GREs that are relevant for that particular tissue. It has been shown by John et al. that this cell type-specificity is predetermined by differences in chromatin landscapes which affect the accessibility of GR to bind to its targets [25].

## Conclusions

In this study we identified over 1,100 GBS in neuronal PC12 cells, the majority of which were unique and exhibited very little overlap with GBS in other cell types. The PC12 unique GBS were located in the vicinity of genes involved in neuronal functions such as axonogenesis, neuron differentiation and neuron development. Moreover, we confirmed that in more than half of tested GBS the most closely located gene was indeed GC-responsive, suggesting that these GBS play a role in GC-dependent transcriptional control. Intriguingly, we found striking differences in the identity of genes near GBS with or without a GRE. GBS containing a GRE were more often located nearby genes involved in general cellular functions such as regulation of cell proliferation and intracellular signaling, while tethering GBS, in which GR is indirectly bound to the DNA via another TF, were more often located near genes involved in neuronal function. Finally, we characterized the motif content of the GBS and identified a number of binding sites for TFs that may represent novel crosstalk partners of GR in neurons, and would vastly expand the repertoire of TFs in the GR interactome. Future studies should focus on confirming the binding of these predicted TFs within the identified GBS and on establishing their role as neuronal crosstalk partners of GR and their relevance in other neuronal cell types.

We conclude that the current ChIP-Seq study in neuronal PC12 cells has provided insight into some exciting new aspects of GR-mediated action of glucocorticoids in a neuronal context, an area which has so far remained in the dark. Understanding GR-signalling in a neuronal context is important given the profound effects of glucocorticoids on neuronal plasticity and consequently on brain function.

## Methods

### Cell culture and harvest

Rat pheochromocytoma PC12 cells were cultured and differentiated for ten days with NGF as described before in collagen-coated culture flasks (75 cm2 and 175 cm2 for mRNA-analysis and chromatin immunoprecipitation (ChIP) respectively; Becton Dickinson) [31]. On the last day of differentiation, the cells were stimulated continuously for 90 minutes or 180 minutes with either 100 nM Dexamethasone (DEX) or ethanol (0.1%) in corticosteroid-depleted medium for ChIP or mRNA analysis respectively. For ChIP, after 90 minutes incubation the protein-DNA interactions were fixed by crosslinking for 10 minutes with 1% formaldehyde (Calbiochem, Darmstadt, Germany), after which they were incubated for 10 minutes with 0.125 M glycine. After discarding the medium, the cells were washed twice with phosphate buffered saline (PBS) containing Phenylmethanesulfonyl fluoride solution (PMSF; Fluka, Steinheim, Switserland). Finally, the cells were collected in PBS containing Protease Inhibitors (PI, Roche, Mannheim Germany). The centrifuged cell pellet was stored at −80°C until sonication. For sonication, the defrosted cell pellets were dissolved in 0.6 ml PI-containing RIPA (0.1% SDS, 1% NaDOC, 150mM NaCL, 10mM Tris pH 8.0, 2mM EDTA, 1mM NaVO3, 1%NP-40, β-glycerolphophate and Na-butyrate) and incubated on ice for 30 minutes. Subsequently, the chromatin was sheared (Bioruptor, Diagenode; 25 pulses of 30 sec., 200 W), resulting in chromatin fragments of 100–500 bp. The sheared chromatin-containing supernatants were stored at −80°C until use in the ChIP-procedure. For the mRNA-analysis (n=6), the cells were harvested after 180 minutes incubation with 100 nM DEX and total RNA was isolated using Trizol reagent (Invitrogen Life Technologies, Carlsbad, USA) according to manufacturer’s protocol.

### ChIP-Seq

For ChIP Sepharose A beads (GE Health care, Uppsala Sweden) were blocked with 1 mg/ml BSA (Biolabs, Ipswich, UK) and 0.2 mg/ml fish sperm (Roche) for 1 hr at 4°C. Three independent ChIPs each were performed on chromatin (60–120 μg per treatment) of the same batch of differentiated cells. Per ChIP the chromatin was precleared by incubation with blocked beads for 1 hr. After preclearing, an input sample was taken to control for the amount of DNA that was used as input for the ChIP procedure. The remaining sample was divided into two samples, each incubated O/N at 4°C under continuous rotation with either 6 μg of ChIP-grade GR-specific H300 or normal rabbit IgG antibody (Santa Cruz Biotechnology, California, USA). Subsequently, the antibody-bound DNA-fragments were isolated by incubating the samples with blocked protein A beads for 1 hr at 4°C. The beads were washed 5 times in 1 ml washing buffer (1x low salt; 1x high salt; 1x LiCl; 2x TE according to Nelson et al. [70]) after which they were incubated with 0.25 ml elution buffer (0.1M NaHCO3; 1% SDS) for 15 min (RT, continuous rotation) to isolate the DNA-protein complexes. To reverse crosslink the DNA-protein interactions, the samples were incubated O/N at 65°C with 0.37M NaCl. RNAse treatment (0.5 μg/250 μl; Roche, Mannheim, Germany was performed for 1 hr at 37°C followed by purification of DNA fragments on Nucleospin columns (Macherey-Nagel, Düren Germany). The immunoprecipitated samples were eluted in 50 μl elution buffer [70]. Half of one ChIP-sample was used for sequencing.

For sequencing, DNA was prepared according to the protocol supplied with the Illumina Genome Analyser GA1. In brief, the DNA fragments were blunted and ligated to sequencing adapters after which the DNA was amplified for 18 rounds of PCR. The DNA was electrophoresed on a 2% Agarose gel, of which a region containing DNA fragments 100–500 bp in length was excised. Subsequently, DNA was isolated from this gel-slice with the Qiagen Gel Extraction Kit. DNA quality was checked on the Agilent Bioanalyser (Waldbronn, Germany). Single end sequencing of the first 36 bp of the resulting DNA library was performed on the Illumina Genome Analyser (Leiden Genome Technology Center, Leiden University Medical Center).

### Peak calling and mapping

The single-end read sequences were aligned to the reference rat genome (RGSC v3.4) using the CLC genomics workbench 3.6.5 (Aarhus, Denmark), according to the default settings which allowed up to 1 mismatch per read or 2 unaligned nucleotides at the ends. Subsequently, DEX-induced peaks were detected using the CLC workbench peak finding algorithm in which the null distribution of background sequencing signal was set for both treatments at 1200 bp and the maximum false discovery rate at 5%. Further settings were left at default. Using Galaxy (http://main.g2.bx.psu.edu/) [37,70,71], Refseq genes in the vicinity of the GBS were determined. As a reference genome Rattus Norvegicus 4 (rn4) was used. Data was visualized by uploading wiggle-files containing the raw ChIP-Seq data on the UCSC genome browser (http://genome.ucsc.edu).

### Motif search

The regions containing the GBS were trimmed to 200bp-width sequences and screened for de novo motifs using MEME [70,72]. The 500 most significant GBS were screened for motifs consisting of 8 to 40 nucleotides. The 15 most significant motifs were given as output. Using TOMTOM [73], the identified motifs were compared against databases of known motifs.

### Comparison of PC12 GBS with other datasets

The genomic regions identified in the PC12 cells were compared to two published datasets consisting of GR-bound genomic regions in human A549 cells [26] and in mouse adipocytes [27]. For this purpose, the significant regions of the published datasets were converted to rat equivalents using the Galaxy website (http://main.g2.bx.psu.edu/) under default conditions. Subsequently, these rat regions were compared to the PC12 GR-bound regions and overlap was calculated using Galaxy [37,71].

### Gene ontology analysis

The nearest genes surrounding the significant GBS were analysed with The Database for Annotation, Visualization and Integrated Discovery (DAVID) v6.7 (http://david.abcc.ncifcrf.gov/home.jsp). As a cutoff, the biological processes (BP) that had a Benjamini-Hochberg p-value <0.05 were considered to be significant. Clustering all the identified GO-terms according to their functional annotation was performed under medium classification stringency (standard setting at DAVID).

### RT-qPCR

RT-qPCR was performed to validate GR-binding to identified GBS using the immunoprecipitated chromatin as input. PCR was conducted using the capillary-based LightCycler® thermocycler and LightCycler ®FastStart DNA MasterPLUS SYBR Green I kit (Roche, Mannheim, Germany) according to manufacturer’s instructions. The primers were designed in NCBI/Primerblast according to the following criteria: (a) PCR product size between 80 and 150 bp; (b) an optimal primer size of 20 bp; (c) an optimal Tm of 60°C; (d) amplicon aimed at the centre of the GBS.

The ChIP PCR signal was normalized by subtracting the amount of nonspecific binding of the IgG antibody in the same sample. This was then calculated as a percentage of the amount of input DNA which was originally included into the ChIP procedure. Known GBS upstream of DNA damaged induced transcript 4 (Ddit4) [56] and Metallothionein 2A (MT2a), served as positive controls for the ChIP. As a negative control, exon 2 of Myoglobin 2 (MB) was amplified. MB is involved in oxygen storage in muscle cells and does not contain a GRE to our knowledge. All selected GBS were measured in three independently performed ChIPs, resulting in 3 measurements per validated genomic location. Normalized data were analysed with GraphPad Prism 5 (trial version 5.00; GraphPad Software, Inc.). An unpaired two-tailed *T*-test was used to assess significant GR-binding. All primer sequences for mRNA and ChIP validation are listed in Additional file 4: Table S4 and Additional file 5: Table S5 respectively.

For mRNA analysis, cDNA was synthesized using the iScript cDNA synthesis kit (Bio-Rad, California, USA), according to manufacturer’s instructions. PCR was conducted as described above. All PCR reactions on cDNA were performed in duplo. The standard curve method was used to quantify the expression differences [74]. cDNA values were normalized against Tubb2a expression levels. Normalized data was analysed with GraphPad Prism 5. The non-parametric Wilcoxon Signed Ranks Test was used to assess significant differential expression of GC-responsive genes. Significance was accepted at a p-value < 0.05.

## Abbreviations

AP-1: Activator Protein-1; ChIP-Seq: Chromatin immunoprecipitation combined with next generation sequencing; Ddc: Dopamine decarboxylase; DEX: Dexamethasone; Gabpa: GA binding protein transcription factor, alpha subunit; GATA1: GATA binding protein 1; GBS: Glucocorticoid receptor binding sites; GC: Glucocorticoid; GR: Glucocorticoid Receptor; GRE: Glucocorticoid Response Element; Kcnab1: Voltage-gated potassium channel subunit beta-1; MB: Myoglobin 2; MT2a: Metallothionein 2A; Narg2: NMDA receptor-regulated gene 2; NCoR: Nuclear receptor corepressor; Nfasc: Neurofascin; NRF2a: Nuclear respiratory factor 2 alpha; Per1: Period circadian protein homolog 1; PMSF: Phenylmethanesulfonyl fluoride solution; Prrx2: Paired related homeobox 2; SEM: Standard error of the mean; SMRT: Silencing Mediator of retinoic acid and thyroid hormone receptor; TF: Transcription Factor; TH: Tyrosine Hydroxylase gene; TSS: Transcription Start Site; VEH: Vehicle Treatment; ZBTB3: Zinc finger and BTB (bric-a-bric, tramtrack, broad complex)-domain- containing 3; Zfp281: Zinc finger protein 281.

## Competing interests

The authors declare that they have no competing interests.

## Authors' contributions

RTdJ was involved in the bioinformatical analysis of the data. JB and DSB were involved in the ChIP procedure. JEW helped with the validation of the ChIP-Seq data. JAEP carried out the cell culture, the ChIP, the experimental validation of the data, performed the data analysis and helped to draft the manuscript. SMvdM and ErdK critically revised the manuscript. NAD conceived and designed the experiments, coordinated the study, was involved in data interpretation and drafted the manuscript. All authors read and approved the final manuscript.

## Supplementary Material

Additional file 1 **Table S1. **list of all 1183 GBS. Click here fo file

Additional file 2 **Table S2.** GO PC12 unique GBS. Click here fo file

Additional file 3 **Table S3.** GO GBS with and without GRE. Click here fo file

Additional file 4 **Table S4.** mRNA primer sequences. Click here fo file

Additional file 5 **Table S5.** ChIP primer sequences. Click here fo file
